# Manufacturing and Functional Characterization of Bioengineered Liver Grafts for Extracorporeal Liver Assistance in Acute Liver Failure

**DOI:** 10.3390/bioengineering10101201

**Published:** 2023-10-16

**Authors:** Victoria L. Nelson, Aron R. Stumbras, R. Noelle Palumbo, Shawn A. Riesgraf, Marie S. Balboa, Zachary A. Hannah, Isaac J. Bergstrom, Christopher J. Fecteau, John R. Lake, John J. Barry, Jeff J. Ross

**Affiliations:** 1 Miromatrix Medical Inc., Eden Prairie, MN 55344, USA; astumbras@miromatrix.com (A.R.S.); npalumbo@miromatrix.com (R.N.P.); sriesgraf@miromatrix.com (S.A.R.); mbalboa@miromatrix.com (M.S.B.); zhannah@miromatrix.com (Z.A.H.); cfecteau@miromatrix.com (C.J.F.); lakex009@umn.edu (J.R.L.); jross@miromatrix.com (J.J.R.); 2 Division of Gastroenterology, Hepatology and Nutrition, University of Minnesota, Minneapolis, MN 55455, USA

**Keywords:** perfusion, decellularization, recellularization, acute liver failure, bioengineered liver, extracellular matrix, human umbilical vein endothelial cells, primary human liver cells

## Abstract

Acute Liver Failure (ALF) is a life-threatening illness characterized by the rapid onset of abnormal liver biochemistries, coagulopathy, and the development of hepatic encephalopathy. Extracorporeal bioengineered liver (BEL) grafts could offer a bridge therapy to transplant or recovery. The present study describes the manufacture of clinical scale BELs created from decellularized porcine-derived liver extracellular matrix seeded entirely with human cells: human umbilical vein endothelial cells (HUVECs) and primary human liver cells (PHLCs). Decellularized scaffolds seeded entirely with human cells were shown to adhere to stringent sterility and safety guidelines and demonstrated increased functionality when compared to grafts seeded with primary porcine liver cells (PPLCs). BELs with PHLCs were able to clear more ammonia than PPLCs and demonstrated lower perfusion pressures during patency testing. Additionally, to determine the full therapeutic potential of BELs seeded with PHLCs, longer culture periods were assessed to address the logistical constraints associated with manufacturing and transporting a product to a patient. The fully humanized BELs were able to retain their function after cold storage simulating a product transport period. Therefore, this study demonstrates the manufacture of bioengineered liver grafts and their potential in the clinical setting as a treatment for ALF.

## 1. Introduction

Acute Liver Failure (ALF) is a life-threatening illness characterized by the rapid onset of abnormal liver biochemistries, coagulopathy, and the development of hepatic encephalopathy. While clinical outcomes for patients with ALF have steadily improved with changes in medical management, the primary therapy for patients with ALF remains liver transplantation, with a 1-year post-transplant survival rate of 91% [1]. However, a recent analysis of data from the Acute Liver Failure Study Group (ALFSG) Registry and the Scientific Registry of Transplant Recipients (SRTR) reported that only 64% of patients listed for transplant received a lifesaving organ. This analysis highlights both the limited availability of livers available for transplantation and the rapid clinical progression of ALF, especially for patients with drug-induced liver injury, with most clinical outcomes determined within 4 days of admission [2].

Various technologies have been explored for providing extracorporeal liver assistance in the treatment of ALF [3,4]. Some of the critical attributes that distinguish these technologies include cell type, cell source, source species, cell mass, bioreactor design, membrane and/or filter attributes, and perfusate. While the use of porcine hepatocytes and human tumor-derived C3A cells has raised safety concerns surrounding immunological response, zoonotic disease transmission, and tumorgenicity, technologies including HepatAssist (Circe) and Extracorporeal Liver Assist Device (ELAD, Vital Therapies) have received regulatory approval for human trials [5,6,7]. Despite promising in vitro and pre-clinical data, disappointing human trial results and failure to achieve commercialization suggest significant limitations remain for extracorporeal liver assist technologies to achieve clinically meaningful hepatic function for the treatment of ALF.

Extracorporeal bioengineered liver (BEL) grafts offer an alternative therapy for patients with ALF as a bridge to transplant or recovery [8]. This extracorporeal strategy circulates patient blood through a metabolically active BEL to provide critical liver support. Clinical translation of this technology has been limited by challenges, including reconstitution of a functional vascular network, engraftment of multiple cell types, defining appropriate culture conditions to ensure liver function and stability, and the ability of BEL grafts to maintain functionality during therapy.

Researchers have previously demonstrated in vitro functionality of BEL grafts created through the recellularization of acellular liver scaffolds [9,10,11,12,13,14,15]. More recently, we have reported on the ability to use perfusion decellularization of porcine livers to remove porcine cellular material and create an extracellular liver matrix capable of recellularization with human cells. We have demonstrated functional revascularization of this matrix after seeding with human umbilical vein endothelial cells (HUVECs) [16,17,18]. This matrix successfully sustained continuous perfusion for up to 20 days without systemic anticoagulation in a porcine heterotopic implant model following HUVEC seeding and culture in an in vitro bioreactor system [17]. More recent work has established methods for isolating, seeding, and culturing primary liver cells into revascularized liver grafts and characterized these bioengineered livers (BELs) for liver-specific functions [15,18].

To avoid immune rejection in pre-clinical models, early BELs described above were seeded with porcine hepatocytes. Here, we describe the manufacture of a bioengineered liver graft using primary human liver cells (PHLCs) containing mainly hepatocytes and the evaluation of its potential as a clinical product.

## 2. Materials and Methods

### 2.1. Porcine Organ Procurement and Whole Liver Decellularization

Whole porcine livers (500 to 700 g) were excised from cadaveric pigs, rinsed with PBS, and flushed with saline. Flushed livers were treated with E-beam prior to decellularization (E-Beam Services, Cranbury, NJ, USA). In preparation for decellularization, the Portal Vein (PV), Suprahepatic Inferior Vena Cava (sIVC), and Inferior Vena Cava (IVC) were cannulated. The cannulated livers underwent perfusion decellularization with 1% Triton X-100 (VWR, Radnor, PA, USA M143) and 0.6% sodium dodecyl sulfate (VWR, Radnor, PA, USA, 0227) through the PV and sIVC at target pressures between 12 and 17 mmHg. The decellularized livers were then disinfected with peracetic acid (PAA; U.S. Water, BI0032–6). The decellularized grafts were washed with phosphate-buffered saline (PBS; Corning, Corning, NY, USA, 21–040-CMX12) and stored.

### 2.2. Evaluation of Viral Inactivation

Evaluation of viral inactivation and clearance was performed using a validated in vitro cell culture infectivity assay (Charles River Laboratory Services, Wayne, PA, USA) in compliance with the U.S. Food and Drug Administration’s Good Manufacturing Practice regulations and Q5A(R2) guidance. In brief, liver tissues were sectioned into 1“by 2” patches, rinsed with 100 mL 0.9% saline, and incubated with 1 mL of virus stock solution for 15 min. Murine Leukemia Virus (MuLV), Pseudorabies (PRV), Reovirus 3 (Reo3), and Porcine Parvovirus (PPV) were selected as model viruses to ensure full coverage of primary virus attributes, including virus genome, envelope classification, size, and shape [19]. After viral spiking, test and reference materials were subjected to representative process treatments or held for analysis, respectively. Viral titers of test and reference materials were determined according to the Spearman-Karber Method to determine the common logarithm for each test volume, achieving a 50% cell culture infectivity dose (TCID50). The viral log reduction value was calculated as the difference between the common logarithm of the total viral load of the reference and test materials.

### 2.3. Process Residuals Testing

Fully decellularized liver grafts were compressed, sampled with a punch biopsy, weighed, and digested prior to assays for SDS, Triton, and DNA residuals. Samples for SDS and triton residuals were vortexed in 15 µL of 20 mg/mL Proteinase K (Qiagen, Hilden, Germany) in 1485 µL Tris-HCL buffer, then digested at 60 °C for 18–24 h. Samples were then assayed for any detectable SDS and Triton through gas chromatography and high-performance liquid chromatography, respectively (Pace Analytical, Minneapolis, MN, USA). Punch biopsies for DNA testing and known DNA standards (Thermo Fisher Scientific, Waltham, MA, USA) were digested and purified using the DNeasy Blood and Tissue Kit (Qiagen, Hilden, Germany), with a slightly modified protocol, and then measured on a Qubit 3.0 Fluorometer (Thermo Fisher Scientific, Waltham, MA, USA). 

### 2.4. BEL Culture and Seeding Overview

**Figure 1:**
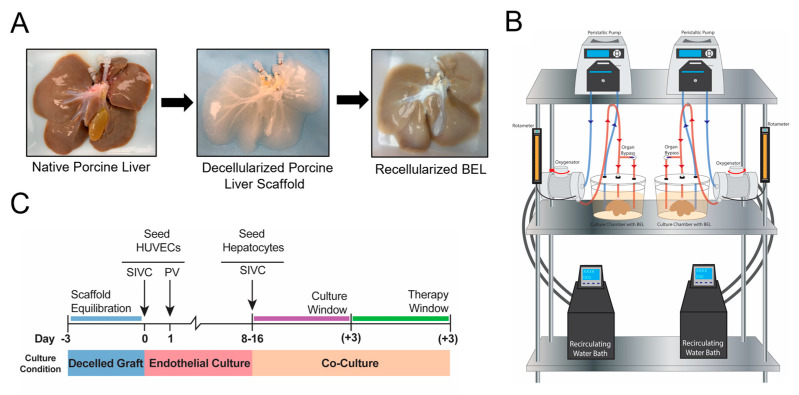
Overview of Manufacturing, Culture, and Therapy Window of BELs. (**A**) Native porcine livers are harvested, decellularized, and recellularized with HUVECs and human hepatocytes. (**B**) Schematic diagram of bioreactor station used to culture BELs. (**C**) Cell culture scheme showing the scaffold equilibration and qualification period, endothelial culture of HUVECs, and bi-culture of HUVECs and human hepatocytes, including the 3-day available therapy window.

Following decellularization, scaffolds were installed into the bioreactor culture station (Figure 1B) and perfused with media for a 3-day qualification period before endothelialization with HUVECs through the supra hepatic IVC and portal vein (Figure 1C). These seeded scaffolds are cultured for 8–16 days to ensure complete HUVEC revascularization prior to primary liver cell seeding isolated from either human or porcine native livers (Figure 1C). BELs seeded with primary porcine liver cells (PPLCs) were cultured for 1 to 3 days after seeding. BELs seeded with primary human liver cells were cultured for 3 days, held in cold storage for 14–16 h, and cultured for an additional 3 days for a simulated therapy window. All BELs were tested and analyzed each day after primary liver cell seeding.

### 2.5. HUVEC Cell Culture and Seeding

Human umbilical vein endothelial cells (HUVECS) (Lonza, Walkersville, MD, USA Cat#C2519A) were cultured at 37 °C and 5% CO_2_ in antibiotic-free media consisting of Endothelial Cell Growth media (R&D Systems, Minneapolis, MN, USA, CCM027) supplemented with a proprietary combination of ingredients optimized for HUVEC culture. Cells were harvested with 0.25% trypsin-EDTA (Thermo Fisher Scientific, Waltham, MA, USA) at 90–100% confluency. Decellularized porcine livers were installed in bioreactors and perfused with species-specific co-culture media (37 °C, 5% CO_2_) for 3 days to confirm the absence of microbial contamination. Co-culture media was a Williams’ E-based medium supplemented with a proprietary combination of ingredients optimized to accommodate both HUVEC and hepatocyte culture. Media optimization differed between porcine and human hepatocytes, and 3D HUVEC culture was initiated in media appropriate for later hepatocyte seeding. HUVECs were collected at passages 5–9, and 1.6 × 10^8^ cells in 180 mL of co-culture media were infused through the sIVC using a syringe. BEL grafts then underwent a 1-h static culture to allow for cell attachment within the scaffold. During this 1-h static hold, temperature and dissolved gas concentrations were maintained in the bioreactor by perfusing culture media through the system while bypassing the organ. After the 1-h static hold, 4.0 × 10^7^ HUVECs in 90 mL culture media were infused into the sIVC with a syringe under continuous flow at 300 mL/min. After an overnight culture, the organ was manually flipped to perfuse on the portal vein. HUVECs were collected and seeded through the portal vein in the same manner described above for the sIVC. Following seeding, grafts were cultured in co-culture media with daily replacement volumes continually adjusted to ensure that glucose remained available throughout the 24-h culture period. Media perfusion into the scaffold was maintained at a maximum 300 mL/min flow rate, with maximum pressures at 30 mmHg.

### 2.6. Porcine Hepatocyte Isolation

Fresh whole livers (450–1000 g) harvested from female pigs and primary porcine liver cells were isolated as previously described [18]. This study was reviewed and approved (No. 040420) by the Institutional Animal Care and Use Committee (IACUC) for MRS/Collagen Solutions. Cell viability and yield were quantified by trypan blue dye exclusion on a hemocytometer, and the final cell pellet was resuspended in a final volume of 1 L using the University of Wisconsin (UW) Belzer solution (Bridge to life, Northbrook, IL, USA).

### 2.7. Human Hepatocyte Isolation

Organs for research to advance medical science were obtained by Donor Network West, NDRI, and Southwest Transplant Alliance and were generously gifted with the consent of the donor or the donor’s next of kin. Liver tissue was flushed via the inferior vena cava, hepatic artery, and portal vein using Lactated Ringers Solution (LRS; Hutchins & Hutchins, Waynesboro, VA, USA). The right and left lobes were resected, and exposed vessels were flushed with cold LRS and cannulated for perfusion. Liver tissue was perfused (10–50 mL/min) with Liver Perfusion Solution I (VitroPrep, Durham, NC, USA) for 12–15 min, followed by perfusion with Liver Perfusion Solution II (VitroPrep, Durham, NC, USA) for 20–40 min. Liver Perfusion Solution II was supplemented with both collagenase MA (3 mg/L; Vitacyte, Indianapolis, IN, USA) and protease BP (2.5 mg/L; Vitacyte, Indianapolis, IN, USA) to initiate digestion. Solutions were not recirculated. Digested tissue was diluted in human hepatocyte isolation medium (HHIM; DMEM with 10% FBS) and sieved (1000, 500, 250, 90 µm). Hepatocytes were enriched by centrifugation (110× *g*, room temperature, 10 min) and washed once with HHIM. Cell viability and yield were quantified by trypan blue dye exclusion on a hemocytometer. The final cell pellet was resuspended in an equivalent volume of UW Belzer solution.

### 2.8. HUVEC and Hepatocyte Characterization

HUVECs were taken from final cell suspension prior to seeding into grafts. Cells were fixed using 2% paraformaldehyde (Electron Microscopy Sciences, Hatfield, PA, USA) and stained for CD31 (BIORAD, Hercules, CA, USA #MCA1738; 1:100 dilution) and CD105 (BIORAD, Hercules, CA, USA #MCA1557; 1:100 dilution) expression. Expression was evaluated by flow cytometry using a BD Accuri C6 Flow Cytometer (BD Biosciences, Franklin Lakes, NJ, USA). PHLC samples were taken from the final cell suspension of freshly isolated PHLCs. Cells were fixed using 4% paraformaldehyde (Electron Microscopy Sciences, Hatfield, PA, USA), permeabilized with Triton X-100 (Sigma, St. Louis, MO, USA), and stored in FACS buffer (1x PBS (Corning, Corning, NY, USA) with 10% BSA (Sigma. St. Louis, MO, USA) and 0.5% NaN_3_ (Ricca, Arlington, TX, USA)). Cells were stained for asialoglycoprotein receptor 1 (ASGR1) expression (antibody from R&D Systems, Minneapolis, MN, USA, #FAB43941R; 1:20 dilution) and analyzed by flow cytometry using an Attune NxT Flow Cytometer (Thermo Fisher Scientific, Waltham, MA, USA). Hepatocytes were identified via size using forward and side scatter and ASGR1 expression as compared to the isotype control (R&D Systems, Minneapolus, MN, USA; IC002R; 1:20 dilution). Data analysis was performed using FlowJo software (BD Biosciences, Franklin Lakes, NJ, USA).

### 2.9. Hepatocyte Seeding

A range of 5B-10B hepatocytes for BEL recellularization was targeted as this has previously been shown to be supportive of native liver function in models of hepatic failure [20,21,22,23,24,25]. Primary liver cells from either porcine or human isolations were allowed to pellet overnight at 4 °C. Following the overnight hold, the supernatant was removed from the pellet, and either 10 × 10^9^ porcine hepatocytes or 5 × 10^9^ human hepatocytes were transferred to cell bags and infused through the sIVC of the re-endothelialized BEL scaffold perfusing at 350 mL/min (typically 12–16 days following the first HUVEC seeding) through repeated syringe injection at a rate of 94 mL/min. Hepatocyte-seeded BELs were then returned to continuous media perfusion through the portal vein with co-culture media at a maximum flow rate of 300 mL/min and maximum pressures of 30 mmHg.

### 2.10. Analysis of Metabolites

Media samples from bioreactors were collected daily and immediately assayed on a CEDEX Bio HT bioanalyzer (Roche, Basel, Switzerland) to determine levels of glucose and ammonia in the culture media. As previously described, measured glucose concentrations were used to calculate daily consumption rates over a 24-h period prior to complete daily media changes and used to determine the level of re-endothelialization and necessary media volume for the following media change [17]. Additional samples were collected daily for quantification of urea, alpha-1 antitrypsin (A1AT), and fibrinogen and stored at −80 °C for analysis. Urea levels were analyzed via commercially available kits following the 5 µL/well protocol from the manufacturer with plate readings at 430 nm after 50 min. A standard curve was serial diluted on each plate using the kit provided standard from 50 mg/dL to 0.78 mg/dL (BioAssay Systems, Hayward, CA, USA DIUR-100). A1AT quantification was performed with a commercially available kit (Abcam, Cambridge, UK, ab189579). The manufacturer’s protocol was modified to analyze all standards and samples in triplicate. Fibrinogen was performed with a commercially available kit (Abcam, Cambrige, UK ab241383). The manufacturer’s protocol was modified to analyze all standards and samples in triplicate.

### 2.11. Quality Testing

Due to the short time between the production of HUVECs and PHLCs and their introduction into the manufacturing process, microbial testing was focused on detecting any contaminants introduced by these starting materials after they were seeded into the decellularized matrix. USP <71> compendial sterility testing was performed post-HUVEC seeding and after the transfer for the simulated cold storage transport, where interim 5 to 7-day reads were evaluated prior to 14-day results. A rapid microbial method (RMM) using a Gram stain test was also performed to aid in real-time detection of microbial contamination 14–24 h post-PHLC seeding. Rapid mycoplasma testing via nucleic acid amplification was performed post-HUVEC seeding and prior to cold storage simulation using aseptically collected spent media. Rapid mycoplasma testing was optimized in-house by modifying the large-scale protocol of a commercially available kit (Thermo Fisher Scientific, Waltham, MA, USA 4460626) using a QuantStudio 5 Real-Time PCR System (Thermo Fisher Scientific, Waltham, MA, USA). Qualification of the method demonstrated a limit of detection that harmonized with European Pharmacopoeia 2.6.7 and aligned with culture-based USP<63> test methodologies.

### 2.12. BEL Ammonia Clearance

Fresh media was exchanged within the bioreactors containing the bi-culture BELs 14–24 h after seeding hepatocytes. As media was perfused through the system while bypassing the organ, ammonium chloride was dosed into the system at a final concentration of 200 µM. The media was allowed to circulate with the dosed ammonium chloride for 10 min before a 0-h sample was collected, and the perfusion was switched to flow ammonium chloride-containing media through the organ inlet, resuming normal media perfusion. Samples were collected after 1 h, and the ammonia levels were quantified on the CEDEX Bio HT Bioanalyzer. Ammonia clearance values were calculated by subtracting the 0-h sample from the 1-h sample.

### 2.13. Data Analysis

Ammonia clearance data was collected in real time. Fibrinogen, A1AT, and urea production data were collected and analyzed from frozen samples and analyzed post-hoc using an unpaired *t*-test for statistical significance between human and porcine grafts. Trends in human grafts were analyzed post-hoc for statistical significance using a repeated measure mixed-effects ANOVA analysis (α < 0.05 deemed significant). Data were compared between days 1–7, 1–3, and 5–7. A1AT was not compared on days 6 and 7 due to an insufficient sample size (*n* = 2) and only compared between days 1–5 and 1–3. Ammonia clearance was compared between the cold storage and day 7 instead of day 5. Ammonia clearance rates were calculated by measuring the difference in ammonia levels between the 0- and 1-h post-bolus timepoints. Data was reported as mean ± one standard deviation where applicable.

### 2.14. Cold Storage and Transport Simulation

For the cold storage study, BEL grafts were removed from bioreactor perfusion after 72 h of culture and transferred to a static transport container. Grafts were flushed with 2 L of cold UW Belzer solution (Bridge to Life, Northbrook, IL, USA) at 300 mL/min. Transport containers were stored in a cooler filled with ice for 14–16 h. After cold storage, BELs were flushed with 2 L of room temperature PBS at 300 mL/min and returned to the bioreactor system, where perfusion was restarted with 4 L of fresh co-culture media. Ammonia clearance was evaluated approximately 10 min after media perfusion began and daily thereafter. Samples were collected for A1AT and fibrinogen quantification daily immediately prior to media changes.

### 2.15. Blood Perfusion Studies

For in vitro blood perfusion studies, each BEL was connected to a circuit consisting of silicone tubing, a pressure transducer (Deltran Utah Medical, Midvale, Utah, USA DPT-100), a peristaltic pump (Cole-Palmer, Vernon Hills, IL, USA 07522-20), and an oxygenator (LivaNova, London, UK, 050703) warmed with a recirculating water bath to 37 °C and receiving 20% O_2_, 5% CO_2_, and 75% N_2_ blended gas. Freshly collected heparinized porcine whole blood was warmed to 37 °C, and the activated clotting time (ACT) was measured (ITC, Hemochron Response, Bedford, MA, USA). A 10 mg/mL solution of protamine sulfate was gradually added to the blood to neutralize heparin until an ACT of 400–600 was reached. Two liters of blood were introduced into the circuit and perfused through the portal vein of the BEL at an initial flow rate of 350 mL/min. Pressures were recorded over 180 min of blood perfusion.

## 3. Results

### 3.1. Decellularization

All porcine livers were successfully harvested and decellularized as described above (Figure 1A). Select decellularized liver graft sections subjected to the in vitro cell culture infectivity assay confirmed viral inactivation and clearance with a log reduction value greater than 6.00 for each model virus (Table 1). Select decellularized liver graft sections subjected to the in vitro cell culture infectivity assay confirmed viral inactivation and clearance with a log reduction value greater than 6.00 for each model virus (Table 1), in accordance with ISO-22442 [19,26]. Removal of porcine cellular material and associated process residues was confirmed using representative lots of decellularized porcine liver scaffolds tested for DNA, triton, and SDS (Table 2). The mean residual DNA in assayed grafts immediately after recellularization was 72 ng/mg (23–116 ng/mg). After a 72 h media perfusion period to mimic the 3-day scaffold equilibration period preceding recellularization, the remaining residual DNA fell below the detectable limit of the assay (Table S1). Because DNA measurements were taken after successful viral inactivation by E-Beam and PAA treatment of porcine livers, any remaining DNA potentially of viral origin is no longer a risk for infection. Residual Triton and SDS were below the limit of detection of 1 ppm for all assayed grafts. These residuals demonstrate that the decellularization process successfully removes porcine cellular material without leaving behind residuals that would harm the subsequently seeded cells.

### 3.2. HUVEC and Hepatocyte Characterization

**Figure 2:**
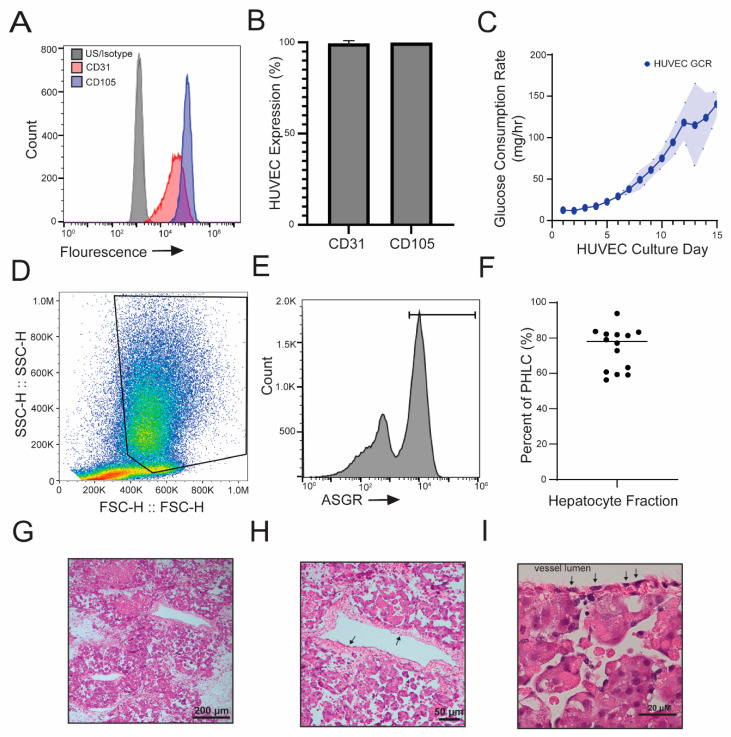
Cellular identification and histology of BELs. HUVECs and PHLCs were characterized prior to seeding and within the graft. HUVECs expression of CD31 and CD105 was evaluated by flow cytometry prior to seeding into the decellularized graft. (**A**) Representative histogram of HUVEC expression of endothelial markers. (**B**) Percentage of HUVECs stained positive for CD31 and CD105 from Figure 1A (*n* = 25). (**C**) Glucose consumption rate during the endothelial culture period of the decellularized scaffold. The shaded area depicts two standard deviations from the mean. (**D**) Flow Cytometry scatter profile of PHLC population. Hepatocytes are indicated based on size and complexity by gated area. Gating is representative. (**E**) ASGR expression within a representative PHLC population. Gate indicates positive expression as compared to appropriate isotype control. The population shown is representative of the PHLC population after isolation. (**F**) Hepatocyte quantification within the BEL population as determined from the scatter plot gating strategy. (*n* = 14) (**G**) H&E showing a cross-section of a BEL recellularized with HUVECs and PHLCs one-day post hepatocyte seeding. (**H**) H&E showing a cross-section of a HUVEC lined vessel in a BEL recellularized with HUVECs and PHLCs. Arrows show representative areas of HUVEC-lined vessels. (**I**) H&E showing a highly magnified cross-section of a BEL recellularized with HUVECs and PHLCs hepatocytes one-day post hepatocyte seeding. Arrows show HUVECs lining the vessel.

To confirm the phenotype of HUVECs prior to seeding into the decellularized porcine scaffold, the expression of endothelial specific markers CD31 and CD105 was assessed by flow cytometry (Figure 2A,B). Over 90% of HUVECs express CD31 and CD105 at the time of seeding (2B). HUVEC expansion after seeding into the decellularized porcine scaffold is indicated by increasing glucose consumption over time (Figure 2C) and histological examination of HUVEC-only BELs collected at different timepoints in the culture period (Figure S1). H&E of HUVEC and PHLC seeded BELs confirm HUVECs are lining the vasculature (Figure 2H,I).

Flow cytometry was also used to evaluate the PHLC population after isolation. PHLCs were evaluated both by scatter plot profile (Figure 2D) and ASGR expression (Figure 2E). Hepatocytes were identified as large cells on the scatter plot, as profiles and marker expression between donors were variable (Figure S2A). The gating demonstrated in Figure 2D is representative of the strategy used. The median value of hepatocytes within the PHLC population is over 75% (Figure 2G), though variability is seen across different donors. PHLCs were seeded into decellularized grafts regardless of flow characterization. The variability observed did not appear to impact function as all donor hepatocytes cleared ammonia when seeded into the HUVEC-lined BEL (Figure S2B). After seeding, hepatocytes engrafted into the parenchyma of the revascularized liver scaffold (Figure 2H,I).

### 3.3. Quality Testing

To confirm the absence of any adventitious agents and microbial contaminants, testing was performed post-HUVEC seeding and again on days 14–16. All lots tested were suitable for use in our manufacturing process as they were determined to be free of microbial contamination, endotoxin, and mycoplasma at each timepoint tested.

### 3.4. Functional Comparison between BELs Seeded with PPLCs and PHLCs

**Figure 3:**
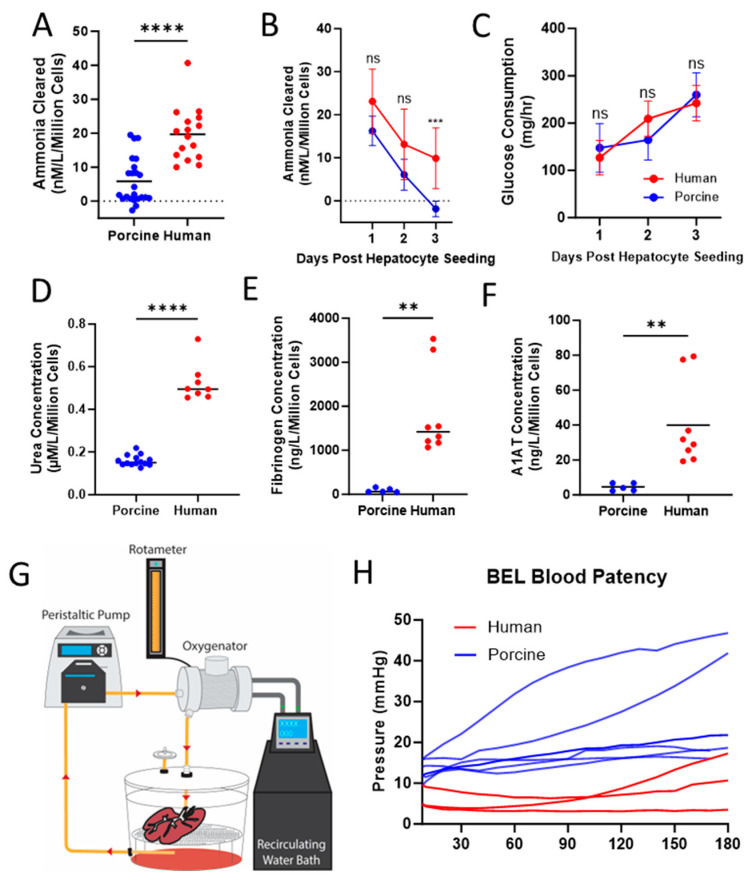
Comparison of Function between Porcine and Human BELs. (**A**) Ammonia cleared from media after one hour post 200 µM bolus to the porcine (blue) and human (red) BEL, data expressed per million cells (*n* = 23 porcine, *n* = 16 human). (**B**) One hour of ammonia clearance was performed daily for 3 days of BEL culture in porcine (blue) and human (red) BELs, with data expressed per million cells (*n* = 5 porcine, 10 human). (**C**) Glucose consumption calculated daily after 24 h of culture for 3 days in porcine (blue) and human (red) BELs (*n* = 24 porcine, 10 human) (**D**) Urea concentration in BEL conditioned media collected at the end of a 24-h culture period, data expressed per million cells (*n* = 14 porcine(blue), *n* = 8 human(red)). (**E**) Fibrinogen production in BEL conditioned media collected at the end of a 24-h culture period, data expressed per million cells (*n* = 5 porcine(blue), *n* = 8 human(red)) (**F**) A1AT production in BEL conditioned media collected at the end of a 24-h culture period, data expressed per million cells (*n* = 5 porcine (blue), *n* = 8 human (red)). (**G**) Schematic showing circuit for blood perfusion studies. (**H**) Porcine (blue) and human (red) BEL patency was measured for 180 min in a blood perfusion circuit (*n* = 6 porcine BEL, 3 human BEL). Statistical significance ** *p* < 0.01, *** *p* < 0.001, **** *p* < 0.0001, and ns = not significant. represent the results of a *t*-test between porcine and human data, and both (**B**,**C**) show results with a false discovery correction.

BELs seeded with either PPLCs or PHLCs were subjected to an ammonia clearance assay the day after seeding. One hour after administration of the ammonia chloride bolus, PHLC-seeded BELs cleared 19.70 ± 7.83 nM per liter of ammonia per million cells in comparison to 5.86 ± 6.73 nM per liter cleared per million cells by BELs seeding with primary porcine liver cells (Figure 3A). Because grafts will need to maintain function to facilitate clinical logistics, we assessed ammonia clearance over 3 days. A subset of BELs seeded with PPLCs that initially cleared an equivalent amount of ammonia as those seeded with PHLCs (16.26 ± 3.42 nM/L/million cells, 23.10 ± 7.50 nM/L/million cells respectively; *p* < 0.05) was evaluated for comparison. After three days of culture, BELs seeded with PPLCs accumulated ammonia during the test with an average clearance of −1.85 ± 1.80 nM/L/million cells. This was significantly less (*p* < 0.0001) than the average amount of ammonia cleared from BELs seeded with PHLCs at the same timepoint (9.90 ± 7.08 µM, Figure 3B).

Despite the differences in cell number at the time of seeding, there was no statistically significant difference in glucose consumption during days 1, 2, and 3 between PPLC BELs and PHLCs BELs (Figure 3C), suggesting that while both PPLC and PHLC BELs have comparable metabolic conditions during culture in the bioreactor, they are not equivalent at the cellular level. BELs seeded with PHLCs demonstrated higher urea (0.525 ± 0.090 vs. 0.160 ± 0.025 µM/L/million cells; *p* < 0.0001), fibrinogen (1831.00 ± 990.70 vs. 90.46 ± 48.28 ng/L/million cells; *p* < 0.01), and A1AT production (39.98 ± 24.43 vs. 4.53 ± 2.18 ng/L/million cells; *p* < 0.01) than BELs seeded with PPLCs one-day after seeding (Figure 3D–F respectively). All BELs tested remained patent and supported continuous flow of oxygenated and temperature-controlled whole porcine blood through a 3-h blood loop assay (Figure 3G,H). When compared to BELs seeded with PPLCs, BELs seeded with PHLCs had lower pressures over the 180 minute assay.

### 3.5. Cold Storage and Transport Simulation

**Figure 4:**
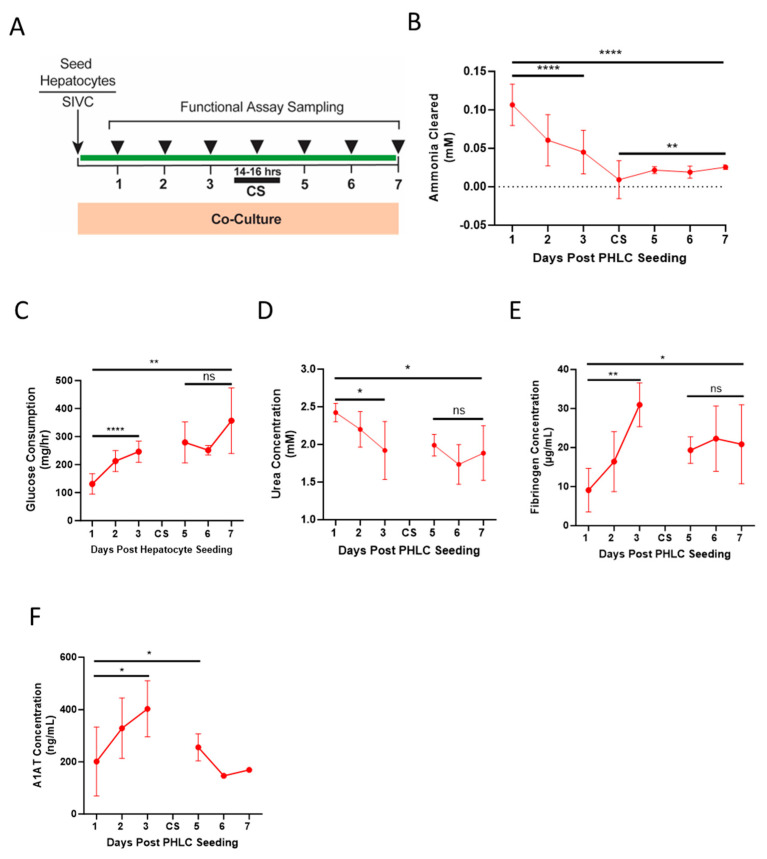
Human BELs display extended function following a cold storage period. (**A**) Schematic diagram showing assay sampling scheme during culture period and 3-day extended hold period during the therapy window after a 14-16 h of cold storage static hold (CS). (**B**) Ammonia cleared from culture media by human BEL after 1-h post-delivery of 200 µM bolus of ammonia, performed daily during the therapy window after the cold storage hold period (*n* = 5–7). Daily measurements of Glucose Consumption (**C**) (*n* = 4–9), Urea (**D**) (*n* = 4–7), Fibrinogen (**E**) (*n* = 4–7), and A1AT (**F**) (*n* = 4–7, *n* = 2 for measurements collected on day 6 and 7) production were quantified in human BELs each day after 24 h of culture during the post cold storage therapy window. Statistical significance * *p* < 0.05, ** *p* < 0.01, **** *p* < 0.0001, and ns = not significant.

To determine the functional stability of the BEL for extended time periods, BELs were cultured for 72 h after PHLC seeding with daily sampling and ammonia clearance assessments. BELs were then subjected to a 14–16 h cold storage period, re-installed into the bioreactor, and cultured for an additional 72 h, again with daily sampling and ammonia clearance assessments (Figure 4A). BELs demonstrated the ability to metabolize ammonia over the initial 72-h culture period. Clearance rates dropped immediately after the cold storage but stabilized and remained consistent after 24 h of bioreactor culture (Figure 4B). Daily glucose consumption, measured over the entirety of the culture period, steadily increased prior to the culture period and stabilized after the cold storage hold, signifying that the cells of the BEL remained viable and metabolically active (Figure 4C). Further evidence of ammonia metabolism is supported by the presence of urea in the bioreactor media at each timepoint measured (Figure 4D). BELs also synthesized proteins demonstrated by the production of both fibrinogen and A1AT. Concentrations of both proteins increased throughout the 72-h culture period post PHLC seeding. Measured levels of these proteins were lower after the cold storage period but were consistent with concentrations recorded 1–2 days after PHLC seeding (Figure 4E,F).

## 4. Discussion

### 4.1. Manufacturing Process Creates a Viral, Residual, and Microbial-Free BEL

One of the underlying safety concerns with xenotransplantation and the use of materials of animal origins is the transmission of pathogenic foreign material, including those that could elicit an immune response in an already critically ill patient. As our only porcine-based material is the decellularized liver scaffold, we addressed this concern by employing a robust viral inactivation strategy that utilized both E-beam irradiation and a PAA treatment, orthogonal modalities that together successfully inactivate and clear a broad range of potential viruses. The model viruses used in this study were selected for their unique characteristics based on their genome, envelope classification, size, shape, and resistance. This demonstrated inactivation and clearance is a distinct advantage over xenotransplantation strategies that incur risks of viral transmission to patients from both known and currently unknown pathogens. Additionally, we verified the removal of potentially toxic residuals as a byproduct of the decellularization process and confirmed the sterility of our recellularization process, signifying that our BELs are safe for patient therapy.

### 4.2. Fully Humanized BELs Demonstrate Higher Liver Specific Function When Compared to Porcine Seeded BELs

BELs seeded with PHLCs consistently demonstrated higher ammonia clearance and protein production the day after seeding when compared with BELs seeded with PPLCs. Additionally, after an analysis of the highest functioning PPLC seeded BELs, those grafts lacked the ability to maintain function after a 3-day culture period with a steady and significant decrease in ammonia clearance function. This work compares human and porcine BELs created with 5 × 10^9^ hepatocytes or 10 × 10^9^ hepatocytes, respectively. We were able to show greater ammonia clearance, urea, fibrinogen, and A1AT production per cell of human hepatocytes as compared to porcine hepatocytes. Because primary human hepatocytes are a limited resource, 5 × 10^9^ human hepatocytes give greater potential to treat multiple ALF patients from one donor liver. Even though a decrease in ammonia clearance over time was evident in BELs seeded with PHLCs, these BELs maintained the ability to clear ammonia on day 3. This data further highlights the potential and benefit of utilizing primary human cells as a therapy for ALF.

BELs seeded with PHLCs demonstrated a small decrease in pressure during the blood loop assay. While differences in seeding cell number or species differences like cell size may play a factor in this outcome, both types of BELs demonstrated a pressure profile compatible with an ELAP therapy. As only fully human BELs will be used in the clinic, future experimentation will be focused on maintaining or improving this pressure profile.

### 4.3. Humanized BELs Demonstrate Function through Simulated Transport and Clinically Relevant Therapy Window

After cold storage to simulate the transport process to a patient, we observed a drop in ammonia clearance, fibrinogen, A1AT, and urea production. This is not unexpected, as cold ischemia is well known to have a negative impact on tissue metabolism [27]. However, the ammonia clearance, urea, and fibrinogen production post-cold storage remained stable for 72 h beyond the transport period, which opens the door for our BEL as a therapy for patients with ALF. We have shown ammonia clearance rates from 107 ± 0.027 µM/h on day one of culture to 26 ± 3 µM/h on day seven of culture. We acknowledge that the results reported here are performed in vitro; however, the range of ammonia clearance provided by our extracorporeal BEL is well within the range of those reported in previous clinical studies that provided extracorporeal ammonia clearance for the treatment of hyperammonemia [28,29]. This is highly encouraging for the successful translation of our technology into the clinical stage. Future studies will examine the ability of BELs, generated through our defined and consistent manufacturing process, to provide therapy in a clinical setting. Experimentation into expanding BEL function after cold storage will allow for longer and more robust therapy in the future.

## 5. Conclusions

Acute liver failure is a rapidly evolving, devasting disease. The high demand and required wait times for a liver transplant put a patient at risk for life-threatening complications. The data presented here establishes a defined and consistent manufacturing process to create functional BELs composed entirely of human cell types as a bridge therapy for patients with ALF. The described decellularization process provides a high degree of confidence in the inactivation and clearance of a broad range of xenotropic viruses. This process also generated very low residual porcine DNA, Triton, and SDS, which favorably supports the recellularization process and minimizes the risk to patients. These recellularization methods enable coverage of the vasculature by the HUVECs and infiltration of the PHLCs into the parenchyma while the bioreactor maintains BEL culture parameters to facilitate consistent cellular growth, resulting in at least 6 days of liver function, including the final 3 days of the therapy window. Demonstrating function over 6 days allows for a recellularized BEL to continue bioreactor culture for up to 3 days if a patient is not ready to receive treatment. The ability of our BEL to remain functional throughout culture, demonstrate activity after cold storage transportation, and provide liver function in a prolonged therapy window points to its potential to provide effective therapy for patients with ALF.

## 6. Limitations

While BELs demonstrated critical hepatic function relevant for the treatment of ALF, there are limitations with this study that may benefit from further experimentation. BEL functionality evaluated through ammonia clearance and protein production was conducted within the in vitro bioreactor system perfused with co-culture media. This system is designed to maintain the metabolic activity of the graft by accurately controlling temperature, dissolved gas, pressure, and flow at appropriate levels while supplying all essential nutrients and cytokines. This in vitro setup may not predict performance in a complex in vivo system perfused with whole blood. While patency testing was conducted with whole blood perfusion, the duration of these studies was limited compared to the intended therapy duration due to degradation of the blood after collection and during patency testing. Further experimentation will focus on bridging the gap between media and whole blood perfusion for in vitro evaluations while advancing the technology toward demonstration of function in vivo.

While the work featured in this study used primary human hepatocytes, future work should examine other liver cell sources, such as human pluripotent stem cells, which are rapidly emerging as a clinically-ready technology in the field of bioengineered organs. As this technology advances into the clinical setting, consideration will be given to the need for and extent of immunosuppression to mitigate adverse patient reactions. Determination of the need and development of such a regimen must account for the unique and transient patient exposure to the cells seeded in the BEL, which will have to be evaluated as part of a clinical study.

## Figures and Tables

**Table 1:** E-beam irradiation and PAA treatment inactivate xenotropic viruses in natural porcine livers.

Virus	Genome	Envelope	Size (nm)	Shape	Resistance	Log Reduction Value
Parvovirus (PPV)	DNA	-	18–24	Icosahedral	V. high	>6.00
Pseudorabies (PRV)	DNA	+	120–200	Spherical	Medium	>6.00
Reovirus 3 (Reo3)	RNA	-	60–80	Spherical	Medium	>6.00
Murine Leukemia virus (MuLV)	RNA	+	80–110	Spherical	Low	>6.00

**Table 2:** Representative residual testing for decellularized porcine liver scaffolds.

Batch	Scaffolds Tested	Mass of Sample Tested (mg)	Residual DNA (ng/mg)	Total Triton (ppm)	Residual Triton (mg/20 g)	Total SDS (ppm)	Residual SDS (mg/20 g)
A	1	76.8	116	<1	<LOD	<1	<LOD
	2	73.6	102	<1	<LOD	<1	<LOD
B	3	82.0	49	<1	<LOD	<1	<LOD
	4	73.2	23	<1	<LOD	<1	<LOD
C	5	81.4	102	<1	<LOD	<1	<LOD
	6	83.9	82	<1	<LOD	<1	<LOD

## Data Availability

Select data presented in this study are available on request from the corresponding author.

## Supplementary Materials

The following supporting information can be downloaded at: https://www.mdpi.com/article/10.3390/bioengineering10101201/s1, Figure S1: HUVEC Growth and Proliferation in 3D culture; Figure S2: Characterization of PHLCs by Flow Cytometry.
