# Supplementary figures and images for: Manufacturing and Functional Characterization of Bioengineered Liver Grafts for Extracorporeal Liver Assistance in Acute Liver Failure
Source: Bioengineering (Basel). 2023 Oct 16;10(10):1201. doi: 10.3390/bioengineering10101201 (PMC10604724; doi:10.3390/bioengineering10101201)

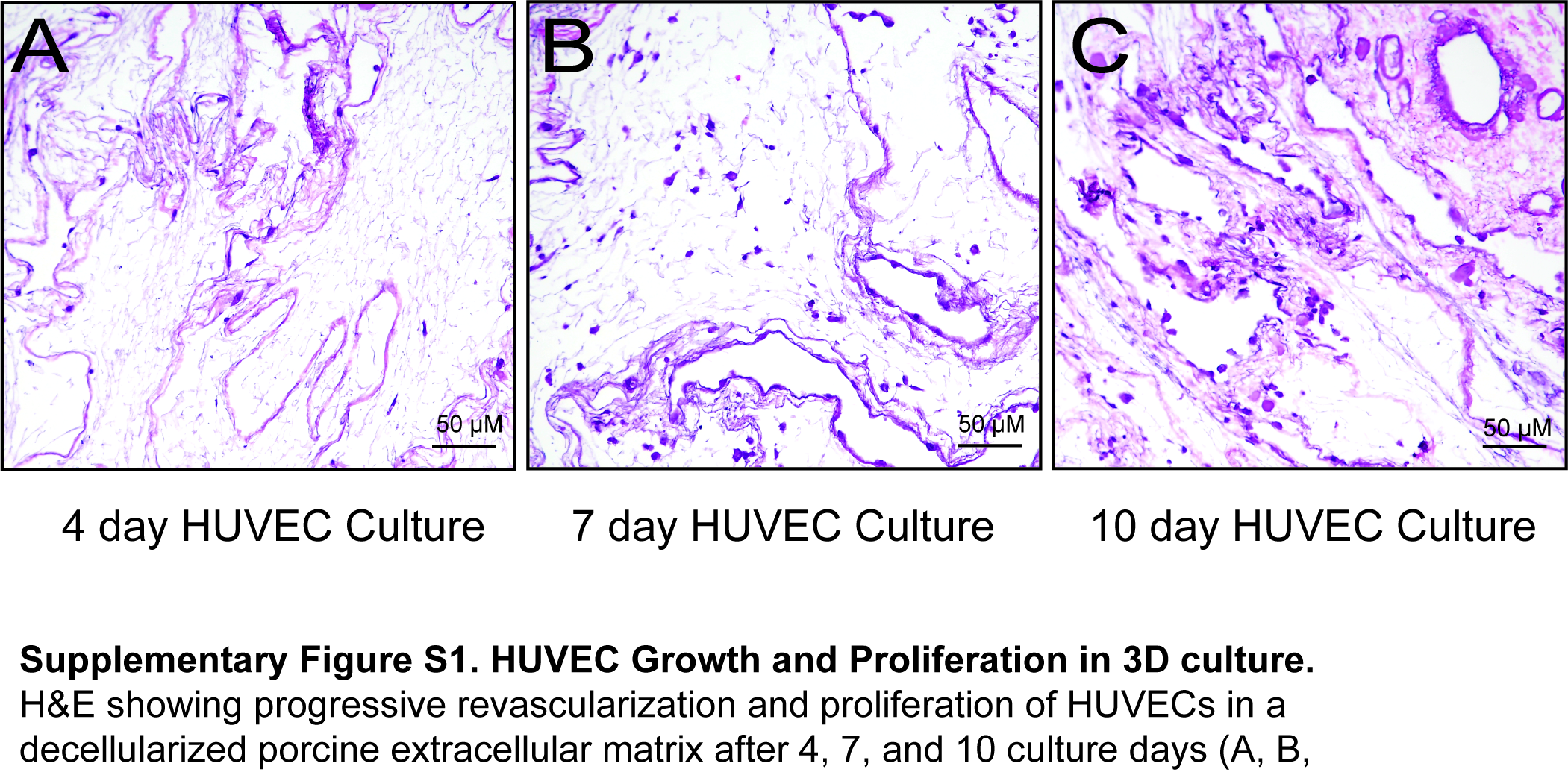

Supplement: Supplementary file 1 [file bioengineering-10-01201-s001.zip › Figure S1.tif]

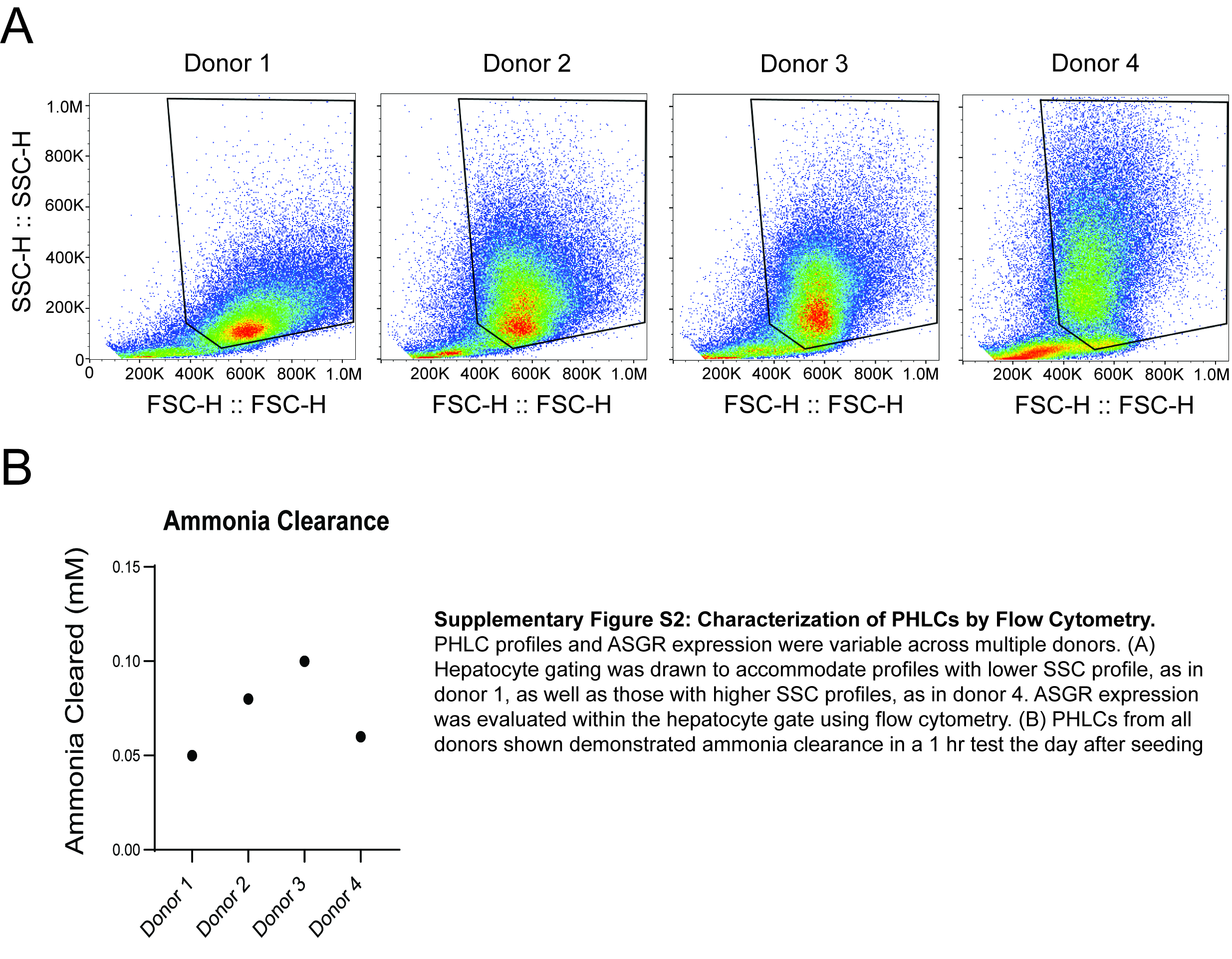

Supplement: Supplementary file 1 [file bioengineering-10-01201-s001.zip › Figure S2.tif]
